# Gasotransmitter Regulation of Ion Channels: A Key Step in O_2_ Sensing By the Carotid Body

**DOI:** 10.1152/physiol.00034.2013

**Published:** 2014-01

**Authors:** Nanduri R. Prabhakar, Chris Peers

**Affiliations:** ^1^Institute for Integrative Physiology and Center for Systems Biology of O_2_ Sensing, Biological Sciences Division, University of Chicago, Chicago, Illinois; and; ^2^Division of Cardiovascular and Diabetes Research, LIGHT, Faculty of Medicine and Health, University of Leeds, Leeds, United Kingdom

## Abstract

Carotid bodies detect hypoxia in arterial blood, translating this stimulus into physiological responses via the CNS. It is long established that ion channels are critical to this process. More recent evidence indicates that gasotransmitters exert powerful influences on O_2_ sensing by the carotid body. Here, we review current understanding of hypoxia-dependent production of gasotransmitters, how they regulate ion channels in the carotid body, and how this impacts carotid body function.

Vertebrate organisms are endowed with a variety of receptors for detecting diverse sensory modalities. Although the molecular basis of this sensory activity is similarly diverse, ion channels commonly play a central role in many transduction pathways, either as primary sensory elements (e.g., for mechanical and thermal sensation) or as downstream effectors (31). The ability of vertebrates to sense hypoxia (decreased O_2_ availability) rapidly is vital for survival and maintenance of homeostatic mechanisms. Carotid bodies are the primary sensory organs for detecting hypoxia, rapidly translating changes in arterial blood O_2_ levels to appropriate physiological responses. Since the discovery of this sensory function, many studies have examined potential hypoxic transduction mechanisms, and the majority of reports support a central role for ion channels in this process, in what has become known as the “membrane hypothesis” for chemotransduction. More recently, emerging evidence also suggests that sensory transduction at the carotid body is uniquely dependent on the O_2_-dependent actions and interactions of gasotransmitters, which act in part via the regulation of ion channels to control hypoxic sensing (67). This article provides a brief review of our present understanding of how O_2_ sensing involves the production of gasotransmitters and their impact on ion channel function in the carotid body.

## Carotid Body: Anatomy, Sensory Capabilities, and Physiological Significance

Carotid bodies are located bilaterally at the bifurcation of common carotid artery into internal and external carotid arteries, allowing them to sense hypoxia (as well as other physiological parameters: CO_2_ and pH) in arterial blood before the stimulus reaches the brain. Carotid body chemoreceptor tissue receives a rich vascular supply, and a branch of the IX cranial nerve (glossopharyngeal), the carotid sinus nerve, provides the sensory innervation. The cell bodies of the carotid sinus nerve reside in the petrosal ganglion.

Ultrastructural studies have provided important insights into carotid body function (46). The chemoreceptor tissue is a collection of type I cell clusters (often termed glomus cells) and type II cells. Although the type I cells are of neural crest origin, type II cells resemble glia and represent a pool of stem cells that can transform into type I cells (56). A large number of studies suggest that type I cells are the initial site(s) of sensory transduction and they work in concert with the closely apposed afferent nerve endings, functioning as a “sensory unit” (36). Sensory discharge under normoxia (arterial blood Po_2_ of ∼100 Torr) is very low but increases dramatically in response to even modest hypoxemia (arterial blood Po_2_ of ∼60 Torr). The sensory response is rapid, occuring within a few seconds after the onset of hypoxia, and the stimulus-response is curvilinear, resembling a mirror image of the O_2_-hemoglobin dissociation curve. Thus the anatomical location, the remarkable sensitivity, and the rapidity with which it responds to hypoxia makes the carotid body an ideal sensory organ for monitoring O_2_ levels in arterial blood.

Well established reflex responses of the carotid body include stimulation of breathing and sympathetic activation by hypoxia (18). The carotid body chemosensory reflex has immense physiological and pathological significance. For instance, carotid body-resected human subjects exhibit attenuated stimulation of breathing during exercise (29, 88), implicating the carotid body reflex in mediating exercise-induced hyperventilation (57, 87). Pregnancy is associated with increased resting breathing and augmented hypoxic ventilatory response (89), and pregnant cats exhibit augmented carotid body response to hypoxia (24), suggesting that carotid chemoreceptors contribute to ventilatory changes during pregnancy. High-altitude sojourns lead to a series of physiological adaptations: ventilatory acclimatization to hypoxia (VAH) represents one such adaptation (63). It is characterized by a progressive increase in baseline ventilation, which ensures adequate oxygen supply, as well as augmented hypoxic ventilatory response (13, 82). Failure to hyperventilate sufficiently during ascent to the altitude leads to development of pulmonary edema (28), and a large body of evidence suggests that carotid body reflex is critical for VAH (reviewed in Ref. 5).

Humans with cardiorespiratory diseases exhibit autonomic dysfunction manifested by elevated sympathetic nerve activity. For example, persistent sympathetic activation and elevated plasma catecholamine levels are hallmarks of sleep-disordered breathing with recurrent apnea, which may underlie cardiovascular co-morbidity (68, 71). Congestive heart failure (CHF) patients also exhibit increased sympathetic nerve activity, which contributes to progression of the disease, and the carotid body chemoreflex contributes to sympathetic activation during CHF (73). People with essential hypertension exhibit an augmented ventilatory response to hypoxia, which has been attributed to enhanced carotid body sensitivity to low O_2_ (79). Indeed, spontaneous hypertensive rats exhibit augmented hypoxic sensitivity of the carotid body (20), and bilateral sectioning of carotid sinus nerves attenuate the magnitude of hypertension in SHRs (1).

Clearly, the ability of the carotid body to sense hypoxia underlies not only acute reflex changes in ventilation but influences cardiorespiratory adaptations to the environment and to pathophysiological maladaptations. The above-mentioned examples of the carotid body's many and varied influences on whole organism physiology and pathophysiology are but a few examples among many, and the reader is referred to a comprehensive recent review for further details (36). Given the widespread influence of the carotid body, it is perhaps unsurprising that the cellular and molecular mechanisms by which O_2_ sensing occurs have been sought for decades. Currently, the majority of opinion supports the membrane hypothesis for chemotransduction, and this is discussed briefly below, partly to provide a framework for understanding more recently discovered roles for gasotransmitters in this complex process.

## The Membrane Hypothesis for Chemotransduction

**FIGURE 1.**
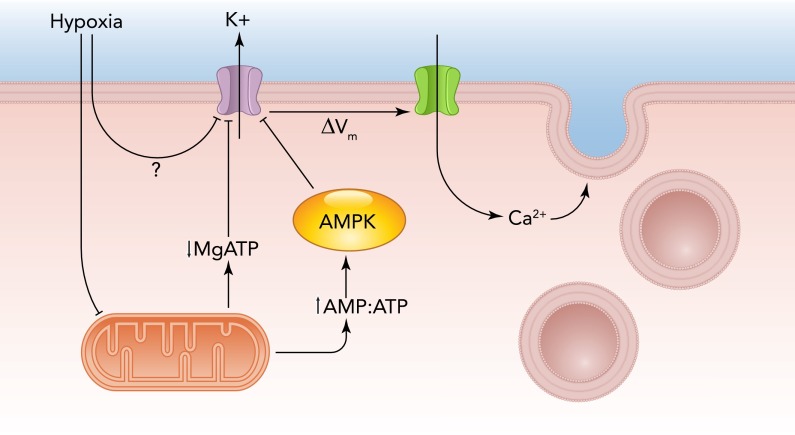
Cartoon of the “membrane hypothesis” for hypoxic chemotransduction in type I carotid body cells Hypoxia inhibits K^+^ channels, leading to type I cell depolarization, opening of Ca^2+^ channels, and hence triggering of neurotransmitters that activate sensory afferent fibers. The mechanism of hypoxic inhibition of K^+^ channels is not fully resolved, but current ideas suggest it may involve inhibition of cytochrome oxidase in uniquely O_2_-sensitive mitochondria, leading to reduced ATP levels even in mild hypoxia. This may lead to channel inhibition either through reduced levels of MgATP or via activation of AMP kinase.

The current “membrane hypothesis” for chemotransduction views ion channels as central to the oxygen-sensing capabilities of type I cells. The concept originated with a publication by Lopez-Barneo et al. in 1988 (41) that described for the first time the dependence of the activity of an ion channel on O_2_ levels. Specifically, the authors described a voltage-gated K^+^ channel in rabbit type I cells and how the activity of this channel declined as O_2_ levels were lowered. This discovery rapidly led to the idea that hypoxia, by closing K^+^ channels, led to depolarization of the type I cell membrane potential. This in turn led to opening of voltage-gated Ca^2+^ channels, and the resultant increase in [Ca^2+^]_i_ triggered neurotransmitter release and consequent excitation of afferent sensory fibers within the carotid sinus nerve, which relay to brain stem neurons that regulate breathing. Subsequently, numerous reports of O_2_-sensitive K^+^ channels in the carotid body and other O_2_-sensing tissues (notably the pulmonary vasculature) appeared rapidly (reviewed in Ref. 61). The simplicity of the hypothesis (FIGURE 1) hides several controversies that engulfed the field for some years after the initial report in 1988. Indeed, researchers were initially distracted significantly because of debate concerning the diversity of different types of K^+^ channels that could be regulated by hypoxia. It is clear now that several different K^+^ channel types can be regulated by hypoxia in type I cells, and, although species differences account for some of this diversity, evidence indicates that more than one O_2_-sensitive K^+^ channel can be found within cells of the same species. Thus, in rabbit and mouse type I cells, O_2_-sensitive K^+^ channels of the Kv3 and Kv4 families have been reported (43). A hERG-like channel has also been reported to influence membrane potential in type I cells of rabbits, although its regulation by acute hypoxia has not been determined (55). By contrast, in rat type I cells, both high-conductance, Ca^2+^-sensitive K^+^ (maxiK) channels (59, 92) and acid-sensitive leak K^+^ channels [TASK-like channels (6)] are well documented as being O_2_ sensitive.

The relative importance of these channels, particularly in terms of their influence on resting membrane potential, has been discussed over many years (60) and has yet to be fully resolved. However, a more fundamental question also remains, namely, what mechanism accounts for hypoxic inhibition of type I cell K^+^ channels? Given the diversity of channel subtypes reported to be O_2_ sensitive, it is perhaps not surprising that a definitive mechanism has not been provided to account for hypoxic channel inhibition (32, 61). The idea that K^+^ channels may themselves act as sensors for molecular O_2_ does not receive much current support (despite a lack of definitive, direct evidence to discount this possibility). Instead, attention has recently turned back to the long-standing belief (reviewed in Ref. 8) that mitochondria in type I cells may act as sensors of O_2_ levels. Evans and colleagues suggested that hypoxia causes a rise in the AMP-to-ATP ratio within type I cells, which leads to activation of AMP-activated protein kinase (AMPK). This kinase then directly influences K^+^ channels within type I cells via phosphorylation, causing their inhibition (FIGURE 1). Thus pharmacological activation of AMPK mimicked the effects of hypoxia on K^+^ channel (maxiK and TASK-like) activity, and an AMPK antagonist reversed effects of hypoxia (91). A more simple explanation for the involvement of mitochondria could be a simple fall of [ATP]_i_: Varas et al. (81) suggested that MgATP regulates TASK-like K^+^ channels and that metabolic inhibition inhibited their activity by reducing [MgATP]_i_ (FIGURE 1).

Regardless of whether mitochondria regulate type I cell K^+^ channels via AMPK activation or via a simple reduction in available ATP, can they really be defined as “O_2_ sensors”? Simplistically, all mitochondria are O_2_ sensors, since sufficiently severe hypoxia will eventually compromise the ability of cytochrome oxidase to catalyse ATP production. However, to suit this role in the carotid body, some explanation is required as to why they should respond to moderate levels of hypoxia that would not affect the functioning of other cell types. Only by satisfying this criterion can we account for a role for mitochondria in the exquisite sensitivity of the carotid body to hypoxia. Two possible explanations might account for this: either *1*) type I cells are extraordinarily metabolically active, consuming so much O_2_ that ATP production is limited, or *2*) type I cells possess mitochondria with a uniquely low affinity for O_2_ such that ATP production is compromised at relatively mild levels of hypoxia. This latter idea, originally hypothesized in 1972 (48), was supported by studies in isolated type I cells some 20 years ago (15, 16), which have most recently been confirmed by Buckler and Turner (8). These authors also demonstrated that mild hypoxia inhibited mitochondrial electron transport and, specifically, inhibited complex IV (cytochrome oxidase activity), compromising ATP production at Po_2_ levels consistent with its ability to inhibit K^+^ channels and raise [Ca^2+^]_i_.

It remains to be determined how mitochondria in type I cells are so specialized for this role of acute O_2_ sensing, and, indeed, other mechanisms accounting for acute hypoxic modulation of K^+^ channels have been proposed [e.g., reactive oxygen species (ROS) derived from mitochondria or NADPH oxidases; modulation via altered glutathione (GSH:GSSG) redox status; for reviews, see Refs. 32, 36]. Nevertheless, the evidence for a “specialized” cytochrome oxidase, considered plausible for decades, is now well supported by evidence in type I cells (8). This advance in our understanding also suggests that the “membrane hypothesis” for chemotransduction is something of a misnomer, since the process of hypoxic inhibition of ion channels cannot be considered as confined to the plasma membrane. More importantly, the wealth of data supporting a central role for ion channels indicate that any agent that can modify ion channel activity in carotid body type I cells is likely to influence chemosensitivity. Accumulating evidence suggests that gasotransmitters can exert their important influences on carotid body O_2_ sensing, at least in part, via modification of ion channels in type I cells.

## Gasotransmitters in Carotid Body Chemotransduction and Ion Channel Modulation

All three major gasotransmitters (NO, CO, and H_2_S) have been reported to exert major influences on the carotid body and its ability to sense O_2_ levels. Unsurprisingly, many of the enzymes associated with cellular generation of gasotransmitters have been identified within the carotid body, but their expression is not always restricted to type I cells. Interestingly, those enzymes required for formation of NO and CO are heme-containing proteins, and their enzymatic activity requires molecular O_2_. Furthermore, like O_2_, both NO and CO are gases and bind to heme [although different heme-containing proteins can discriminate between these gases (2, 47, 80)], and most if not all of the biological actions of NO and CO are coupled to activation of heme-containing proteins (75). Therefore, NO and CO can be considered to resemble O_2_ in some respects. By contrast, H_2_S shares more commonality with hypoxia since it promotes a reduced cellular milieu. Emerging evidence, detailed below, suggests that NO and CO are inhibitory, whereas H_2_S is an excitatory gasotransmitter in the carotid body. All three gases exert at least some of their effects via ion channel modulation, yet their effects are not uncontested and remain to be fully resolved.

### Nitric Oxide

NO is produced during conversion of L-arginine to L-citrulline, a reaction catalyzed by a family of enzymes called NO synthases (NOS), which require O_2_ for their activity. Three isoforms of NOS have been identified, including neuronal (nNOS/NOS1), endothelial (eNOS/NOS3), and inducible (iNOS/NOS2) (75). nNOS and eNOS are Ca^2+^ dependent and constitutively expressed, whereas the inducible isoform is expressed in response to a variety of stimuli, including hypoxia, and Ca^2+^ is not required for its activity. All three NOS isoforms contain heme prosthetic groups that bind O_2_. The apparent Kms of the three isoforms of NOS are summarized in Table 1. Among these, nNOS is highly sensitive to O_2_, as evidenced by a high Km, suggesting that even modest reductions in O_2_ concentrations will result in a significant loss of its enzyme activity. Indeed, there is a linear relationship between O_2_ concentration and nNOS activity over the entire physiological range (2). Hypoxia decreases NOS activity in a concentration-dependent manner in the carotid body (70), and NOS activity is Ca^2+^ dependent, suggesting that NO comes from constitutively expressed NOS isoforms, presumably nNOS, in this organ (70).

**Table 1:** The affinity of nNOS, eNOS, iNOS, and HO-2 for O_2_

Apparent *K*_m_ for O_2_	
Protein	K_m_, μM
nNOS	350
iNOS	130
eNOS	4
HO-2	80

Expression of nNOS is not evident in glomus cells but is restricted to nerve fibers innervating these cells (9, 70, 84). NOS blockers (l-NNA or l-NAME) stimulate and NO donors inhibit carotid body sensory activity (11, 70, 78, 85). Furthermore, neuronal NOS knockout mice exhibit markedly augmented hypoxic ventilatory response, which is initiated by the carotid body (34). Stimulation of the cut end of the carotid sinus nerve causes depression of carotid body sensory nerve activity, suggesting that the sensory response to hypoxia is regulated by an efferent inhibitory pathway (17, 50, 51), most likely mediated by NO derived from nNOS located in efferent nerve endings (9, 85).

eNOS is expressed in blood vessels of the carotid body (84). In contrast to nNOS knockout mice, eNOS knockout mice exhibit attenuated hypoxic ventilatory response and modest hyperplasia of glomus cells, and these effects are attributed to local hypoxia caused by vasoconstriction resulting from eNOS deletion (35). iNOS, on the other hand, is not evident in the carotid body under basal conditions but is expressed in response to prolonged hypoxia (14), the significance of which remains to be studied. Thus available evidence suggests that O_2_-dependent generation of NO from nNOS is a physiological inhibitor of the carotid body activity involving the efferent inhibitory pathway.

**FIGURE 2.**
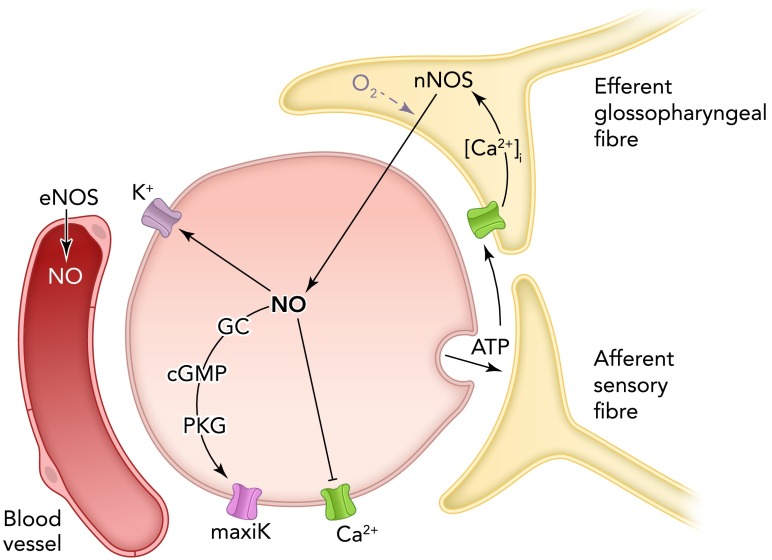
NO as an inhibitory gasotransmitter NO produced in efferent neurons can inhibit L-type Ca^2+^ channels and activate K^+^ channels; either effect will suppress type I cell excitability and transmitter release. In the case of the maxiK channel, NO activation is believed to occur via activation of the guanylate cyclase/cGMP/protein kinase G pathway. Under hypoxic conditions, release of the excitatory transmitter ATP can excite afferent fibers but can also activate Ca^2+^ influx into efferent neurons, thereby activating nNOS and generating NO in a negative feedback pathway.

Summers et al. (76) demonstrated that NO selectively inhibited voltage-gated L-type (and not N-type) Ca^2+^ currents in rabbit type I cells, providing a simple and direct mechanism to account for the inhibitory effects of NO on carotid body hypoxic sensitivity. Interestingly, this important effect of NO was not mediated by cGMP (76). Silva and Lewis (74), by contrast, demonstrated that the NO donor SNAP augmented maxiK channel activity, providing an alternative (or additional) mechanism to account for NO suppression of carotid body activity. This effect was inhibited by a protein kinase G inhibitor, implicating the involvement of cGMP, a finding that contrasts strikingly with earlier studies, which suggested that cyclic nucleotides were not involved in hypoxic chemoreception, at least at the level of maxiK channel inhibition in the rat (26). However, the findings of Silva and Lewis are consistent with the observation that reduced NO bioavailability accounts for suppression of BK channel activity in type I cells from rabbits with experimental chronic heart failure [CHF (38)]. In CHF rabbits, type I cell expression of nNOS is reduced, but adenoviral transfection of the nNOS gene restored maxiK activity (39). Interestingly, Nurse and colleagues have provided compelling evidence that NO is released from efferent terminals of glossopharyngeal neurons as a result of Ca^2+^-dependent activation of nNOS within the terminals in response to Ca^2+^ influx triggered by activation of P2X receptors by ATP released from type I cells themselves. This paracrine response leads to type I cell hyperpolarization (10, 52), presumably arising from K^+^ channel activation, which, as with above-mentioned mechanisms, could account for NO suppression of hypoxic chemosensitivity. Thus the interaction of NO with multiple ion channels (summarized in FIGURE 2) can account for its negative feedback control of carotid body output.

### Carbon Monoxide

In the late 1960s, Lloyd et al. reported that brief inhalation of CO gas eliminated hypoxia-induced hyperventilation (the hallmark reflex initiated by the carotid body) in human subjects (40). Since CO has greater affinity for hemoglobin than O_2_, yet inhibited the hypoxic ventilatory response, it was proposed that carotid body activation by hypoxia involves an unidentified de-oxy conformation of a heme protein(s). This concept has yet to gain further credibility, but the study initiated an extensive period of study of the possible significance of the effects of CO on chemoreception. Current opinion remains divided.

CO is generated during degradation of heme by heme oxygenases, and this reaction requires molecular O_2_ (44). Two isoforms of heme oxygenase have been identified; a constitutively expressed HO-2 and an inducible HO-1 (44). Unlike nNOS, HO-2 is expressed in glomus cells but not in nerve fibers or type II cells, whereas HO-1 expression is not evident under basal conditions (69). The HO-2-heme complex has low affinity for O_2_ (Ref. 47; Table 1), but hypoxia decreases CO generation in the carotid body (66). Blockade of HO by zinc-protoporphyrin-9 stimulates, whereas low concentrations of CO inhibit carotid body activity (69). Although type I cell neurosecretory response to hypoxia is unaltered in HO-2 knockout mice (54), these mice do exhibit markedly elevated baseline carotid body sensory activity and an augmented sensory response to hypoxia (66), suggesting that CO generated by HO-2 is an inhibitory gasotransmitter.

**FIGURE 3.**
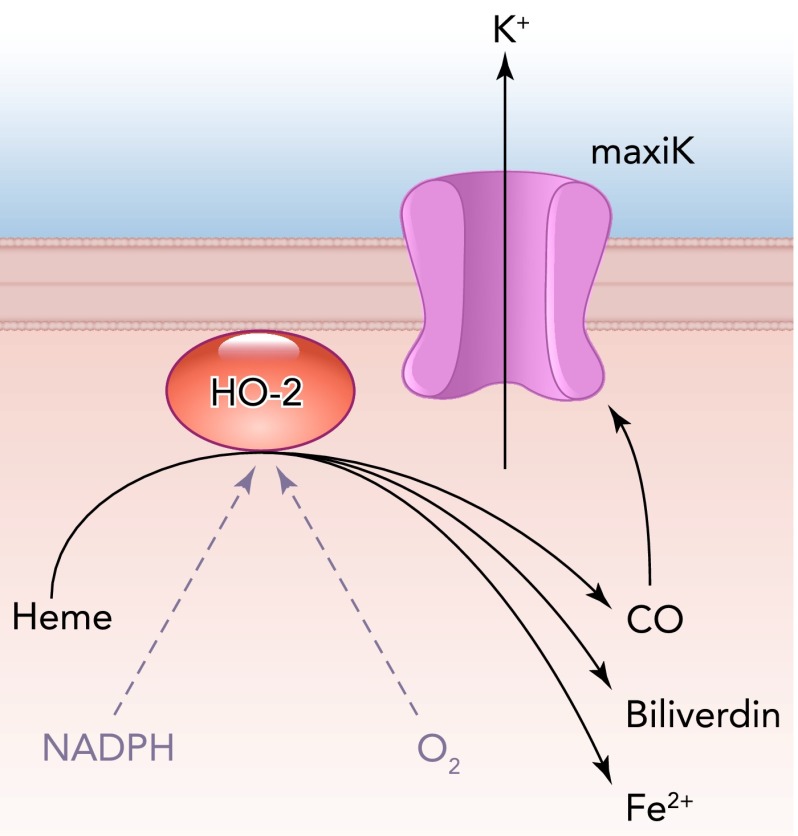
Heme oxygenase-2 is closely associated with maxiK channels, and under normoxic conditions generates CO from heme, using NADPH and O_2_ as cofactors CO activates maxiK channels, preventing cell depolarization. Under hypoxic conditions, tonic production of CO is inhibited, leading to channel closure, which could lead to depolarization, transmitter release, and sensory afferent excitation.

The idea that CO might influence carotid body chemosensitivity via modulation of ion channels has been supported for many years. Perhaps the first study to address this directly came from Lopez-Lopez and Gonzalez (42), who demonstrated that hypoxic inhibition of K^+^ currents in rabbit type I cells could be reversed by CO, leading to their suggestion that “CO interacts with the O_2_ sensor, replacing O_2_ and preventing the inhibition of the K^+^ current” (42). At that time, no molecular basis for O_2_ sensing was apparent, and the concept that K^+^ channels themselves served as direct O_2_ sensors was still an exciting possibility. Subsequent single channel studies from Lopez-Lopez and colleagues (72) confirmed and extended these observations, and concluded that maxiK regulation by O_2_ was membrane delimited and involved a closely associated heme-like protein. Such a proposal proved insightful when it emerged that HO-2 could co-immunoprecipitate with recombinant maxiK channels, suggesting that in native tissues these two proteins were closely associated (90). Provision of HO-2 substrates (NADPH, heme, and O_2_) augmented maxiK channel activity, and withdrawal of O_2_ alone (i.e., hypoxia) greatly reduced channel activity. Furthermore, CO was able to mimic the effects of HO-2 substrate provision and augment maxiK channel activity several-fold. The demonstration that this system could also be reproduced with native maxiK channels in membrane patches excised from rat type I cells led to the proposal that the O_2_ sensitivity of HO-2 activity in type I cells, by regulating maxiK activity, accounted for carotid body hypoxic chemosensitivity (90) (FIGURE 3). However, such an influential role for CO could not be confirmed in HO-2^−/−^ mice (54), in which hypoxia was capable of evoking quantal catecholamine release (which is dependent on cell depolarization and Ca^2+^ influx) in a manner identical to wild-type mice. This may not be particularly surprising, since there are marked differences between the expression and physiological activity of different K^+^ channels in mice and rats (and indeed other species), and indeed the importance of maxiK channel activity even within the rat is still debated (60, 61). Thus the role of maxiK channel activation by CO as a physiological means by which CO acts as an inhibitory gasotransmitter remain to be resolved.

The above-described effects of NO and CO on carotid body function indicate that their levels are high under normoxia and both gases inhibit afferent sensory nerve activity. It was therefore proposed that O_2_-dependent production of NO and CO keeps baseline sensory activity low under normoxia and that hypoxia-evoked increases in sensory activity conceivably arose due to reduced formation of NO and CO, thereby removing their inhibitory influence on sensory activity (64, 67). It should be noted that reports have been published indicating that exogenous application of large doses of NO donors (30) as well as high concentrations of CO (Pco_2_ of ∼320 Torr) stimulate carotid body activity (4). However, such effects of supra-physiological levels of these gases are likely to arise from their interactions with mitochondrial electron transport (86) and do not necessarily negate the proposed inhibitory roles for endogenous NO and CO on the carotid body sensory activity.

### Hydrogen Sulfide

Cystathionine β-synthase (CBS) and cystathionine γ-lyase (CSE) are the two major enzymes first discovered to catalyze the biosynthesis of H_2_S (21, 83). Type I cells express CBS (19, 37, 77) as well as CSE (49, 62). In contrast to NO and CO, H_2_S levels are low in normoxia in the carotid body but increase in response to hypoxia in a stimulus-dependent manner (62). Similar increases in H_2_S levels are also seen in isolated type I cells challenged with hypoxia (45). Hypoxia-evoked H_2_S generation was absent in CSE knockout mice and in rats treated with propargyl glycine (PAG), an inhibitor of CSE (62), suggesting that CSE is the primary source of hypoxia-induced H_2_S generation. H_2_S donors stimulate carotid body sensory activity in a concentration-dependent manner, and the time course and magnitude of such responses resemble those evoked by hypoxia (37, 62). Crucially, the carotid body sensory response to hypoxia was markedly attenuated in CSE knockout mice and in rats treated with PAG, and this was accompanied by a strikingly reduced neurosecretory response to hypoxia from individual type I cells (45, 62). Although aminooxyacetic acid (AOAA) and hydroxylamine (HA), purported inhibitors of CBS, also attenuate carotid body sensory responses to hypoxia (37), a recent study reported that both these compounds are more potent inhibitors of CSE than CBS (3). This notwithstanding, these collective pharmacological and genetic approaches strongly suggest that CSE-derived H_2_S is a major physiological contributor to carotid body stimulation by hypoxia.

**FIGURE 4.**
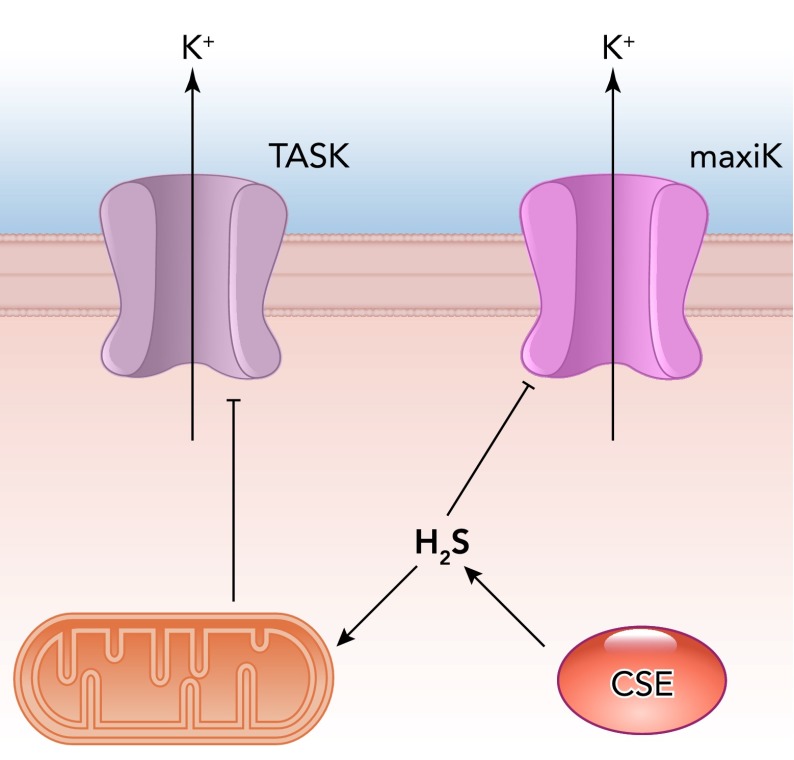
Hydrogen sulfide inhibits both known O_2_-sensitive K^+^ channels in rat type I cells: maxiK and TASK-like channels Inhibition of maxiK channels may be direct, but inhibition of TASK-like channels may be a nonphysiological effect arising from mitochondrial inhibition.

The ability of type I cells to generate H_2_S endogenously has prompted workers to investigate whether type I cell ion channels are modulated by this gasotransmitter. Li et al. (37) were the first to report H_2_S inhibit maxiK channels in the glomus cells mimicking hypoxia (FIGURE 4). Telezhkin et al. (77) confirmed that H_2_S inhibited maxiK channels of rat type I cells and extended their studies to show that human recombinant maxiK channels were similarly inhibited via a mechanism that was distinct from the effects of CO. Thus, although these gasotransmitters interact importantly to determine overall carotid body excitability (see below), they can also act independently, via maxiK channel regulation, to influence type I cell activity. H_2_S has been shown to raise [Ca^2+^]_i_ (45). This is consistent with maxiK channel inhibition, if this channel contributes to the determination of type I cell resting membrane potential (60), but this is not universally accepted (e.g., Ref. 22). Recently, Buckler also demonstrated that H_2_S, like hypoxia and other chemostimuli, could raise [Ca^2+^]_i_ because H_2_S inhibited TASK-like channels in rat type I cells (FIGURE 4), causing membrane depolarization and subsequent voltage-gated Ca^2+^ entry (7). His study concluded that H_2_S acted to inhibit TASK-like channels via inhibition of oxidative phosphorylation but speculated that this was unlikely to be physiologically important since the levels of H_2_S exogenously applied to exert such effects were supraphysiological. A similar argument has been proposed by others (25). However, the data obtained from CSE^−/−^ mice, detailed above, argue strongly that endogenous H_2_S is important, even essential, for hypoxic chemotransduction, and others suggest that it also serves a major role in other O_2_-sensing sytems (53). It is possible that mitochondria-mediated H_2_S regulation of TASK-like channels is not of physiological importance, but H_2_S inhibition of maxiK channels observed in excised patches (77) suggests a possible direct modulation may well account for (or contribute to) the physiological actions of this gas. Clearly, there remains much to resolve concerning the role of this excitatory gasotransmitter in the carotid body as well as in other O_2_-sensing systems.

## Evidence for Interactions Between Inhibitory and Excitatory Gasotransmitters

Peng et al. examined potential interactions between CO and H_2_S in the carotid body (62). They found that an HO inhibitor markedly increased basal H_2_S levels under normoxia and, conversely, that a CO donor inhibited H_2_S generation by hypoxia. Remarkably, these effects were absent in CSE knockout mice (62). These findings lead to the suggestion that the low sensory discharge during normoxia is due to the inhibitory influence of CO on CSE resulting in suppressed H_2_S generation, whereas reduced CO generation during hypoxia relieves the inhibition on CSE, leading to elevated H_2_S levels, which contribute to sensory excitation. Thus biochemical interactions between inhibitory (CO) and excitatory (H_2_S) gasotransmitters constitute a key step in the sensory transduction at the carotid body. It is plausible that physiologically significant interaction could occur at the level of key ion channels (e.g., maxiK) that are known to be modulated by these gases (33).

## Current Limitations and Future Directions

It has been proposed that the curvilinear and temporal response of the carotid body to hypoxia arises from the actions and interactions of sensory and downstream proteins, working in concert in what has been termed a “chemosome” to account for the overall chemoreceptor response to low O_2_ (65). From the evidence reviewed here, it is likely that interactions between the enzymes generating gasotransmitters act in concert with ion channels (particularly K^+^ channels) in type I cells constitute important components of this “chemosome.” In this scenario, the low affinity for O_2_ of NOS and HO-2 confers exquisite sensitivity of the carotid body to even modest levels of hypoxia, whereas interactions of gasotransmitters with K^+^ channels contribute to rapidity of sensory excitation by hypoxia. However, as seems traditional in the field of carotid body chemoreception, controversy hangs over many important issues, and many questions remain before a consensus can be reached on any of these issues. Key issues/questions include the following.

*1*) The functional roles of key ion channels in type I cells. As discussed above, both maxiK and TASK-like channels are O_2_ sensitive in rat type I cells. Both are sensitive to gasotransmitters, but modulation of which channel by which gasotransmitter is physiologically important? Moreover, is the regulation of these or other K^+^ channels by gasotransmitters also physiologically significant in species other than rat?

*2*) Are mitochondria influenced by gasotransmitters? Current evidence indicates type I cell mitochondria are of central importance to O_2_ sensing but also that gasotransmitter activity is crucial to chemoreception. Do physiological levels of gasotransmitters influence mitochondrial function or act by separate, parallel pathways to shape carotid body output?

*3*) To what extent do gasotransmitter interactions influence carotid body chemoreception? For example, although it is established that CO can suppress H_2_S production, it is also known that in other systems CO can, for example, stimulate NO formation (12, 27).

*4*) Do gasotransmitters act via posttranslational modification of ion channels or indeed other target proteins? *S*-nitrosylation by NO or sulfhydration by H_2_S are recognized as target protein modifications of global importance in cell signaling (23, 58), yet evidence of their influence in carotid body chemoreception is not yet established.

Answers to these and many other questions require significant additional study before we can account fully for the influence of gasotransmitters on ion channel activity and the importance of such regulation to carotid body O_2_ sensing. Progress is limited not only by the technical difficulties associated with studying such a small organ (particularly with regard to protein biochemistry) but also by current difficulties associated with quantifying intracellular levels of these biologically important gases. However, answers to the issues are needed not only to satisfy academic curiosity: since heightened carotid body sensitivity to hypoxia has been implicated in the autonomic dysfunction associated with the most prevalent cardiorespiratory diseases such as sleep apnea, CHF, and hypertension, it is also of clinical importance to determine how gasotransmitters and their interactions with ion channels in the carotid body are altered in these disease states.
